# Severity of Onion Tearing and Chemosensory Sensitivity: A Preliminary Investigation

**DOI:** 10.1002/lio2.70452

**Published:** 2026-05-25

**Authors:** Mehmet K. Mahmut, Rachel S. Herz, Anabel Bormann, Susanne Weise, Lok Hei Pang, Arianna Soncini, Thomas Hummel

**Affiliations:** ^1^ Interdisciplinary Clinic of Smell and Taste, Department of Otorhinolaryngology Technische Universität Dresden Dresden Germany; ^2^ Food and Fragrance Lab, School of Psychological Sciences, Macquarie University Sydney Australia; ^3^ Department of Psychiatry and Human Behavior Alpert Medical School, Brown University Medical School Providence Rhode Island USA; ^4^ Department of Otorhinolaryngology, Head and Neck Surgery Faculty of Medicine, The Chinese University of Hong Kong Hong Kong China; ^5^ Department of Medicine and Surgery University of Parma Parma Italy

**Keywords:** chemosensation, nasal patency, olfactory function, onion tears, psychophysical testing, subjective testing, trigeminal sensitivity

## Abstract

**Objectives:**

Involuntary tearing is an annoyingly common consequence of cutting onions. Little is known about who experiences so‐called “onion tears” and to what extent they experience them. Our aim with this study was to explore whether the degree of onion tearing is a phenotypic predictor of olfactory function and trigeminal sensitivity, assessed by both self‐report and psychophysical measures of chemosensory function.

**Methods:**

A total of 1001 participants (598 females; *M*_age_ 44 years) completed four subjective ratings of nasal function (subjective smelling ability, nasal sensitivity, trigeminal sensitivity, and nasal patency), three psychophysical tests (odor identification, AmmoLa intensity, and odor lateralization), and answered questions about the severity of their tearing while cutting onions.

**Results:**

Results revealed that higher levels of onion tearing were associated with better self‐rated olfactory ability, nasal breathing, and higher sensitivity to nasal stinging/burning. No statistically significant relationship was found between the psychophysical tests of olfactory ability and degree of onion tearing. Performance on all measures was also analyzed across three different age brackets (18–35; 36–55; 56+ years) and yielded mixed findings related to subjective ratings of trigeminal sensitivity and olfactory function.

**Conclusion:**

Clinically, there is yet to be a universally accepted measure to determine subjective chemosensory sensitivity, and in particular, trigeminal sensitivity. Despite inconsistencies between the subjective and objective chemosensory tests, our findings suggest that asking someone about the severity of their tearing while cutting onions may be a useful addition to standard testing assessing chemosensory and trigeminal sensitivity.

**Level of Evidence:**

3.

## 1 Introduction

The experience of chopping onions through a haze of prickling tears is a common annoyance for many people, be they professional chefs or at‐home cooks. This tearing is caused by the release of the lachrymatory factor propanthialoxide from piercing the onion's flesh [1] though recent research indicates that the drops released from piercing the onionskin can be reduced by using a sharper knife [2]. Contact of this compound with the conjunctiva causes stinging, irritation, and reflexive tears that aim to flush out the chemical from the eyes [3]. Tearing can also be produced by mechanical and cold temperature stimulation of the conjunctiva [4].

The trigeminal nerve innervates various irritation‐type sensations in the face, including sneezing and tearing from chopping onions. Beyond irritation, the trigeminal nerve is strongly involved in our perception of smell [5]. Almost all odorants activate the trigeminal nerve to some degree; phenyl ethyl alcohol and hydrogen sulfide are among the few exceptions [6]. During high levels of stimulation, the sensations produced from concomitant trigeminal‐olfactory activity range from cooling (e.g., produced by menthol), warming (e.g., produced by cineole), to burning (e.g., produced by ammonia). However, most odorants elicit trigeminal responses at milder levels and reinforce the location of the scent in space [7].

Trigeminal activation has been shown to increase olfactory function. This was recently demonstrated by Badran et al. who found that after 30 min of electrical stimulation to the trigeminal nerve, subsequent threshold detection for guaiacol was significantly decreased compared to sham stimulation (electrode placement with no current) [8]. These findings indicate that in the absence of an odorant, electrical stimulation of the trigeminal nerve alone can increase subsequent olfactory sensitivity. It has also been shown that using trigeminally‐stimulating odorants (e.g., eucalyptus) increases the efficacy of “trigeminal training” for improving subjective nasal breathing [9]. Moreover, several studies have shown that individuals with anosmia have decreased trigeminal sensitivity [10] and the loss of trigeminal function dramatically reduces olfactory function [11]. Of particular interest, many patients with olfactory dysfunction report a noticeable reduction in tears when cutting onions after olfactory loss [12].

No research to date has explored subjective individual variability in onion tearing and its relationship to chemosensory sensitivity and the present study was conducted to examine this relationship. Participants completed both self‐report ratings and psychophysical measurements of olfactory and trigeminal function and answered questions regarding the impact of cutting onions on their level of eye tearing. We hypothesized that individuals who reported higher levels of tearing while cutting onions would also demonstrate better olfactory function and higher trigeminal sensitivity.

## 2 Methods

This study included individuals who visited the Deutsches Hygienmuseum–Dresden (“German Museum of Hygiene – Dresden”) from November 2023 to September 2024. Exclusion criteria were breastfeeding, people aged under 18 years, absence of legal capacity, and pregnancy. Written informed consent was obtained from all participants. The study was approved by the Ethics Committee at the Medical Faculty of TU Dresden (application number: BO‐EK‐343082023) and was conducted in accordance with the Declaration of Helsinki.

Assessment of participants included a questionnaire to record demographic information and medical history, focusing on documenting the presence of concomitant chronic rhinosinusitis, allergies, and smoking history. Chemosensory function of participants was assessed with self‐report ratings and psychophysical tools. A total of seven measures were administered (detailed below) by researcher AB, which took ~20 min per participant.

### 2.1 Measures and Procedure

#### 2.1.1 Subjective Measures

Four subjective measures of olfactory were administered and are detailed below.

##### 2.1.1.1 Self‐Rated Olfactory Ability

Participants were asked to rate their ability to smell on a Visual Analogue Scale (VAS) ranging from 0 to 100 (0 = no olfactory function, 100 = excellent olfactory function).

##### 2.1.1.2 Self‐Rated Nasal Patency

Nasal breathing for both nostrils, individually and combined, was also assessed on a subjective VAS from 0 to 100 (0 = completely blocked, 100 = very wide nasal passages).

##### 2.1.1.3 Trigeminal Sensitivity Self‐Report Survey

Participants were asked to self‐rate their general nasal sensitivity to sensations of stinging/burning from 0 to 3 (0 = not at all to 3 = very), and to complete the Trigeminal Survey [13]. The Trigeminal Survey consists of eight items measuring subjective nasal breathing and trigeminal sensitivity (e.g., “Pungent or burning smells make me cough or sneeze”; “In winter, cold air in my nose is extremely unpleasant”) with four response options ranging from 0 (does not apply at all) to 3 (absolutely true). A total score was calculated by summing the scores to each question and ranged from 0 to 24, with higher scores indicating a higher trigeminal sensitivity.

##### 2.1.1.4 Onion Sensitivity Questions

Finally, as part of a larger survey, all participants were asked about their eye sensitivity to cutting onions. Specifically, participants were asked if their eyes water a lot when they cut onions with four response options ranging from 0 (does not apply to me at all) to 3 (absolutely true). This variable is termed “Onion Tearing Sensitivity” and higher scores indicate higher sensitivity.

Partway through the study, two additional questions were added and completed by 452 participants, which were the following: (1) How often do you cut fresh onions per month (variable name “Onion Cutting Frequency”; 0 = never; 1 = once per month; 2 = several times per month; 3 = several times per week) and (2) Has the watering of your eyes changed in the last 10 years with three response options (variable name “Changes in Eye Watering”; response options: yes, more; no change; yes, less).

#### 2.1.2 Psychophysical Measures

Three psychophysical measures of olfaction were administered and are detailed below.

##### 2.1.2.1 Three Item Sniffin' Sticks Identification Test

Participants were tested with a three‐item Sniffin' Sticks Identification test (i.e., cinnamon, banana, and fish; [14]). Each of the three odors are presented with four plausible responses with only one response being correct. A total score was calculated by summing the number of correct responses.

##### 2.1.2.2 Intranasal Trigeminal Sensitivity With AmmoLa Probe

To test intranasal trigeminal function, an ammonium stimulus (AmmoLa probe, DEVESA Dr. Reingraber GmbH & Co. KG, Ribnitz–Damgarten, Germany) was presented to participants, who then rated the intensity of the stimulus on a VAS from 0 to 100. The probe is a lipstick‐sized device that is presented ~2 cm under the participant's nostrils.

##### 2.1.2.3 Odor Lateralization Test of Trigeminal Sensitivity

A lateralization test, where blindfolded participants identify which nostril an odor is nearest, was administered using pure eucalyptol. It comprised 20 trials (10 presentations to each nostril), randomizing left–right nostril presentations with an interstimulus interval of ~10–20 s [15]. A total lateralization score was calculated by summing the correct responses; scores ranged from 0 to 20, with higher scores reflecting higher trigeminal sensitivity.

### 2.2 Procedure

The measures listed above were presented in the following order: Self‐rated olfactory ability, three‐item Sniffin' Sticks Identification Test, self‐rated nasal patency, intranasal trigeminal sensitivity with AmmoLa probe, trigeminal sensitivity self‐report survey, odor lateralization test of trigeminal sensitivity, and onion sensitivity questions.

### 2.3 Data Analysis

Data were analyzed using the Jamovi 2.6.44 software for Windows [16, 17]. Descriptive analyses were performed on the demographic information and dependent measures. Skewness and kurtosis checks indicated all continuous variables were normally distributed, except for the AmmoLa intensity and onion tearing sensitivity scores. For normally distributed data, a one‐way analysis of variance (ANOVA) was conducted to test group differences on the various olfactory measures. The Mann–Whitney *U*‐test and Friedman test were used for non‐normally distributed data. All statistical tests were two‐sided, with *p* < 0.05 considered statistically significant. To compare multiple groups, the non‐parametric Kruskal–Wallis test was used, and significant effects were subsequently tested using the Dwass–Steel–Critchlow–Fligner (DSCF) test for pairwise comparisons between groups.

## 3 Results

### 3.1 Participant Characteristics

A total of 1001 participants completed the study, 598 females and 396 males. Seven participants did not indicate their sex. The mean age was 44.1 years (range 18–86, SD = 16.8; 10 people did not provide their age); 829 participants indicated they were non‐smokers, 124 had a history of allergy, 30 indicated they had chronic rhinosinusitis, and four participants were affected by chronic rhinosinusitis and a concomitant allergy.

### 3.2 Subjective Tests

Table 1 provides descriptive statistics for both the subjective and psychophysical tests participants completed. For the “Onion Tearing Sensitivity” question, 37% reported that it was partly true for them, 36% reported that it was absolutely true for them, 19% reported that it was slightly applicable to them, and 8% reported that it was not at all true for them. For the “Onion Cutting Frequency” question, 38% indicated they cut onions several times per month, 34% indicated they cut onions several times per week, 14% indicated they cut onions once per month, 9% indicated they never cut onions, and 5% indicated they cut onions daily. Finally, for the “Changes in Eye Watering” question, 71% reported that there had been no change over 10 years, 19% reported that there had been a reduction, and 10% reported that there had been an increase.

**TABLE 1:** Descriptive statistics for subjective and psychophysical chemosensory tests.

Chemosensory tests	*N*	Mean (SD)	Min–max
Subjective			
Subjective smelling ability	997	72.3 (20.7)	0–100
Nose sensitivity to stinging/burning	1001	2.34 (0.86)	0–4
Trigeminal survey score	1001	9.1 (4.29)	0–24
Nasal breathing—both nostrils	996	70.5 (20.08)	0–100
Psychophysical			
Odor identification	1001	2.63 (0.60)	0–3
AmmoLa intensity^a^	1001	97 (89–99)	0–100
Odor lateralization	548	14.4 (3.26)	0–20

^a^ AmmoLa data was not normally distributed, so the median and interquartile range is reported.

### 3.3 Psychophysical Tests

As displayed in Table 1, the mean odor identification test score was 2.63 (SD = 0.60), the mean AmmoLa intensity rating was 91.5 (SD = 12.7), and the mean odor lateralization test score was 14.4 (SD = 3.26).

### 3.4 Exploring Olfaction and Trigeminal Function Based on Onion Tearing Sensitivity

To explore whether other onion tearing sensitivity was associated with the subjective and/or psychophysical tests administered, we conducted further analyses (see Table 2). First, based on the question regarding onion tearing sensitivity, participants were divided into two groups. The lower onion tearing sensitivity group (*n* = 264) comprised those who selected 0 or 1 (i.e., not at all or slightly applicable, respectively), and the higher onion tearing sensitivity group (*n* = 733) comprised individuals who selected 2 or 3 (i.e., partly true or absolutely true, respectively).

**TABLE 2:** Comparison of onion tearing sensitivity groups on subjective and psychophysical chemosensory tests.

Chemosensory tests	Lower onion tearing sensitivity		Higher onion tearing sensitivity		Group differences test *p* value
	Mean (SD)	*n*	Mean (SD)	*n*	
Subjective					
Subjective smelling ability	69.2 (21.6)	263	73.5 (20.21)	730	0.004
Nose sensitivity to stinging/burning	2.2 (0.84)	264	2.4 (0.87)	733	< 0.001
Trigeminal survey score	7.9 (4.16)	264	9.5 (4.25)	733	< 0.001
Nasal breathing—both nostrils	69.4 (20.62)	262	71 (19.86)	730	0.29
Psychophysical					
Odor identification	2.6 (0.62)	264	2.6 (0.58)	733	0.73
AmmoLa intensity^a^	97 (89–99)	264	97 (10)	733	0.53
Odor lateralization	14.4 (3.29)	117	14.5 (3.24)	430	0.82

*Note:* *p* value reflects the results of a one‐way analysis of variance (ANOVA).

^a^ Mann–Whitney *U*‐test was performed given data was not normally distributed, so the median and interquartile range is reported.

Given known sex differences in olfactory sensitivity [18, 19], the first analysis was to determine whether there were any sex differences between the Onion Tearing Sensitivity groups we created. To do so, a chi‐squared test was used to evaluate the distribution of sex across the two onion tearing sensitivity groups created. This revealed a significant difference in the distribution between the two groups with 127 females and 134 males in the lower onion tearing sensitivity group and 467 females and 262 males in the higher onion tearing sensitivity group (*χ* ^2^_(1)_ = 19.00, *p* < 0.001). Within the lower onion tearing sensitivity group, compared to males, females had significantly higher subjective smelling ability (*t*(258) = 2.60, *p* = 0.01), nasal breathing—both nostrils (*t*(257) = 2.48, *p* = 0.014), Trigeminal survey score (*t*(259) = 2.56, *p* = 0.011), and AmmoLa intensity scores (*W*(253.5) = 2.69, *p* = 0.008).

Similarly, within the higher onion tearing sensitivity group, compared to males, females had significantly higher subjective smelling ability (*t*(724) = 3.36, *p* < 0.001), nasal breathing—both nostrils (*t*(724) = 3.73, *p* < 0.001), nose sensitivity to stinging/burning (*t*(727) = 2.00, *p* = 0.046), Trigeminal survey score (*W*(589) = 4.00, *p* < 0.001), and AmmoLa intensity scores (*W*(409) = 3.04, *p* = 0.003).

Compared to those in the lower onion tearing sensitivity group, participants in the higher onion tearing sensitivity group reported significantly higher scores on all the self‐rated tests (i.e., subjective smelling ability, sensitivity to burning/stinging and total trigeminal survey score), except for the nasal breathing (both sides) measure (see Table 2). However, there were no significant differences between the lower and higher onion tearing sensitivity groups on any of the psychophysiological tests (see Table 2); odor identification, AmmoLa intensity, and odor lateralization. Finally, participants in the Lower Onion Tearing sensitivity group were slightly older than participants in the higher onion tearing sensitivity group (46.7 ± 17.2 years vs. 43.3 ± 16.6 years; *t*(985) = 2.84, *p* = 0.005).

### 3.5 Exploring Olfactory Ability and Sensitivity as a Function of Age

Given the established findings that olfactory and trigeminal sensitivity decreases with age [15, 20], we explored the impact of age across all the tests we employed (see Table 3). Participants were allocated to three age groups based on the age stratification used in previous research [21]: Group A was composed of participants aged 18–35 years, Group B from 36 to 55 years, and Group C, those aged 56 years and older. To compare the three age groups, a one‐way ANOVA was conducted with all the variables (except the AmmoLa intensity) with Games–Howell post hoc tests due to unequal variances between the tests. The AmmoLa intensity scores were non‐normally distributed, and thus were analyzed using the non‐parametric Kruskal–Wallis test and the DSCF test for post hoc pairwise comparisons.

**TABLE 3:** Performance on self‐report and psychophysical tests by age group.

Chemosensory tests	Age group (years)	*N*	Mean (SD)	Min–max	Age group difference test; *p* value
Self‐rated					
Subjective smelling ability	(A) 18–35	331	73.57 (18.15)	7–100	NS
	(B) 36–55	382	72.37 (21.03)	0–100	
	(C) 56+	274	71.02 (22.89)	4–100	
Nose sensitivity to stinging/burning	(A) 18–35	333	2.23 (0.82)	0–4	B > A; *p* < 0.05
	(B) 36–55	384	2.4 (0.87)	0–4	
	(C) 56+	274	2.38 (0.91)	0–4	
Trigeminal survey score	(A) 18–35	333	8.41 (4.13)	0–20	C > A; *p* < 0.001
	(B) 36–55	384	9.07 (4.15)	0–21	C > B; *p = 0*.054
	(C) 56+	274	9.87 (4.54)	0–24	
Nasal breathing—both nostrils	(A) 18–35	332	69.34 (21.02)	0–100	NS
	(B) 36–55	383	70.23 (19.54)	15–100	
	(C) 56+	271	71.58 (19.57)	10–100	
Onion tearing sensitivity	(A) 18–35	331	2.05 (0.91)	0–3	C < B; *p* < 0.05
	(B) 36–55	383	2.07 (0.89)	0–3	
	(C) 56+	273	1.88 (0.98)	0–3	
Psychophysical					
Odor identification	(A) 18–35	333	2.74 (0.51)	0–3	C < A; *p* < 0.001
	(B) 36–55	384	2.66 (0.56)	0–3	C < B; *p* < 0.001
	(C) 56+	274	2.45 (0.70)	0–3	
AmmoLa intensity^a^	(A) 18–35	333	96 (15)	6–100	
	(B) 36–55	384	97 (9)	0–100	C < B; *p* < 0.05
	(C) 56+	274	96 (8.75)	0–100	
Odor lateralization	(A) 18–35	171	14.84 (3.31)	4–20	C < A; *p* < 0.05
	(B) 36–55	210	14.46 (3.15)	7–20	
	(C) 56+	161	13.96 (3.33)	5–20	

*Note:* A = 18–35 years old; B = 36–55 years old; C = 56+ years old. *p* value reflects the results of post hoc analyses after a one‐way analysis of variance (ANOVA) except for the AmmoLa intensity test.

Abbreviation: NS, non‐significant within group differences.

^a^ Mann–Whitney *U*‐test was performed given data was not normally distributed, so the median and interquartile range is reported. Only significant post hoc, pairwise comparisons are presented.

**FIGURE 1:**
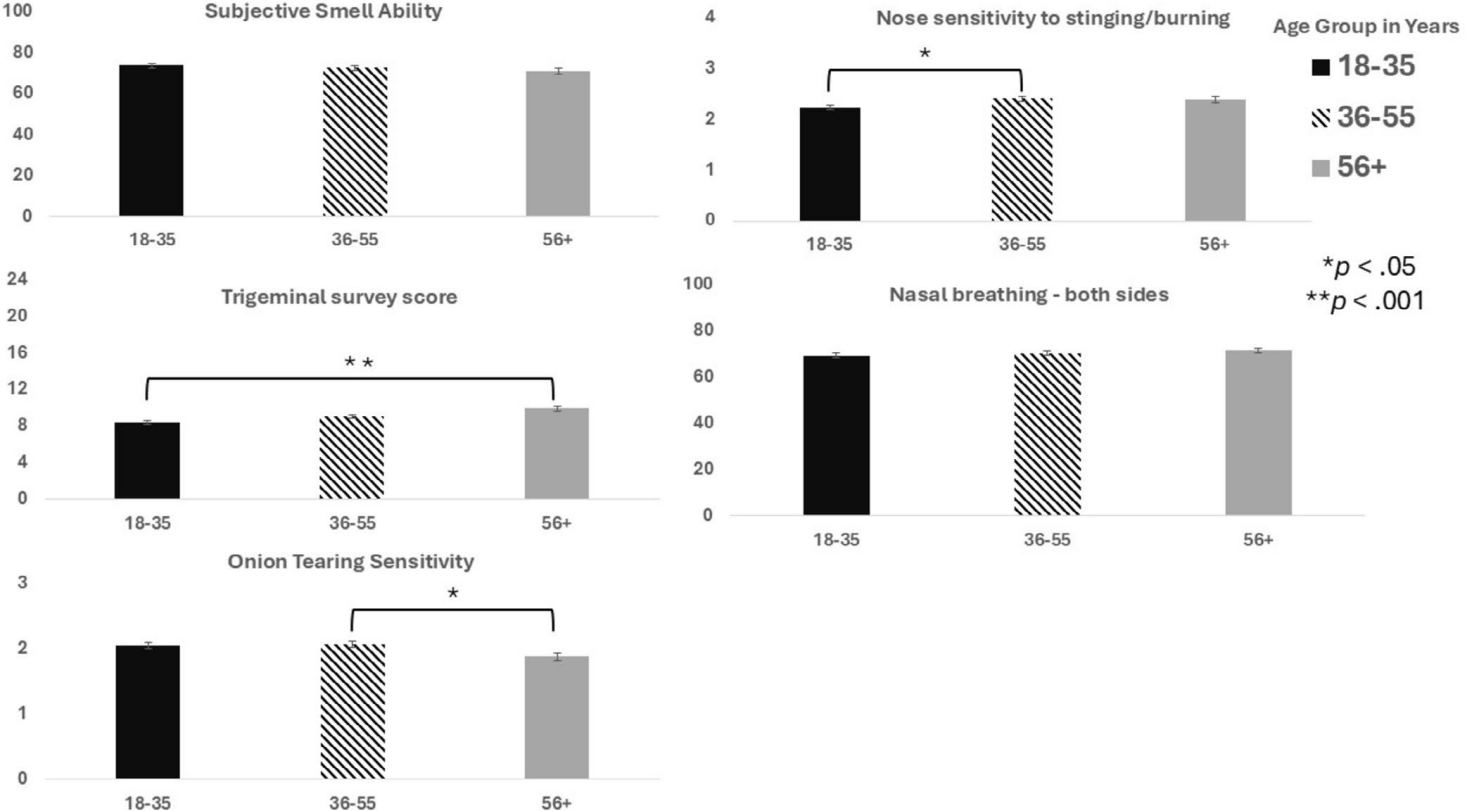
Bar graphs showing subjective chemosensory tests results. Error bars reflect the standard error of the mean. Subjective smell ability was measured on the visual analogue scale ranging from 0 to 100 where higher scores mean better ability. The total trigeminal survey score ranged from 0 to 24 where higher scores mean higher sensitivity. Onion tearing sensitivity was measured using a scale ranging from 0 (does not apply to me at all) to 3 (absolutely true). The response to the nose sensitivity to stinging/burning question was measured from 0 to 3 (0 = not at all to 3 = very). Responses to the nasal breathing—both sides question was assessed on a subjective VAS from 0 to 100 (0 = completely blocked, 100 = very wide nasal passages).

As detailed in Table 3 and summarized in Figure 1, post hoc analyses revealed that subjective smelling ability scores decreased slightly with age, whereas nasal breathing from both nostrils scores increased with age; however, the age group differences were not statistically significant (*F*_(2618)_ = 1.12, *p* = 0.32; *F*_(2627)_ = 0.93, *p* = 0.40, respectively). In contrast, self‐rated sensitivity to nasal stinging/burning was significantly higher for 36–55 year‐olds compared to 18–35 year‐olds (*p* < 0.05; mean difference: 0.17; 95% CI: 2.14–2.49). Additionally, those aged 56+ years had significantly higher trigeminal survey scores compared to 18–35 year‐olds (*p* < 0.001; mean difference: 1.46; 95% CI: 7.97–10.4). Finally, while there was a significant main effect for age group for the Onion Tearing Sensitivity question (*χ* ^2^_(2)_ = 6.23, *p* = 0.044), the DSCF post hoc analysis revealed no significant differences between the three age groups, although participants in the 56+ years old group bordered on having statistically lower Onion Tearing Sensitivity compared to those in the 36–55 years age group (*W* = 3.24, *p* = 0.057).

**FIGURE 2:**
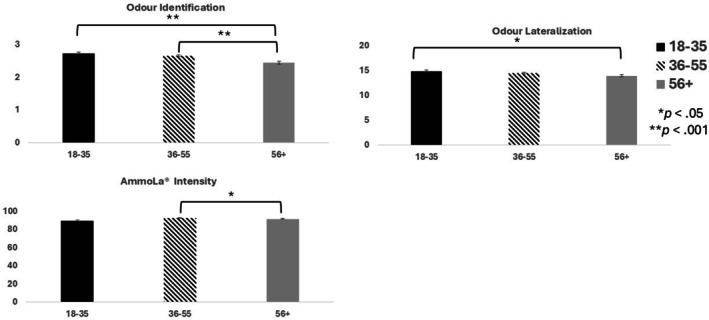
Bar graphs showing psychophysical chemosensory tests results. Error bars reflect the standard error of the mean. Odor identification scores could range from 0 to 3, where higher scores mean better olfactory performance. AmmoLa intensity was measured on a visual analogue scale (VAS) ranging from 0 to 100, where higher scores reflect higher intensity. Odor lateralization scores ranged from 0 to 20, with higher scores reflecting higher trigeminal sensitivity.

Table 3 also details the psychophysical test results, and the summary of these results are presented in Figure 2. Table 3 shows odor identification scores were significantly lower for those in the 56+ years age group compared to those in the 36–55 years age group (*p* < 0.001; mean difference: 0.21; 95% CI: 2.54–2.61) and in the 18–35 years age group (*p* < 0.001; mean difference: 0.29; 95% CI: 2.37–2.54). Additionally, AmmoLa intensity scores were significantly lower in the 56+ years age group compared to the 36–55 years age group (*W* = 3.32; *p* < 0.05). Finally, odor lateralization decreased slightly with age, with the 56+ years age group demonstrating significantly lower scores than the 18–35 years age group (*p* < 0.05; mean difference: 0.88; 95% CI: 13.4–15.3).

## 4 Discussion

The aim of the study was to explore the relationship between onion tearing sensitivity and various subjective and psychophysical chemosensory tests. Our research has shown for the first time that higher levels of self‐reported onion tearing are associated with significantly higher scores on self‐rated tests of chemosensory function. This relationship was not observed with psychophysical tests.

The results revealed that the majority of people (73%) reported experiencing tears while cutting onions. To explore sensitivity to onion tears further, we divided the sample into those who experienced relatively less and those who experienced relatively more onion tears. We found that those who experienced higher onion tearing sensitivity also self‐reported significantly better subjective smelling ability, significantly higher nasal sensitivity to stinging/burning, and significantly higher scores on a trigeminal sensitivity survey. However, there were no significant group differences between those with lower and higher onion tearing sensitivity on the three psychophysical tests we employed: odor identification, AmmonLa intensity, and odor lateralization. These findings are consistent with previous research demonstrating low correlations between subjective olfactory ability ratings and psychophysical olfactory tests [22]. Such inconsistencies occur frequently and have been reported across many domains, such as subjective and physiological ratings of sexuality [23], intensity of experienced emotions [24], and anxiety symptoms [25].

Consistent with normative data [26], older age was associated with significantly lower odor indication ability. When comparing participants' self‐ratings based on age groups (i.e., 18–35, 35–55, and 56+ years), the results were mixed. For example, for both the nasal stinging/burning question and trigeminal survey total score, we found that being in the older age group was associated with significantly higher olfactory sensitivity, whereas for onion tearing sensitivity being in the older age group was associated with significantly lower sensitivity. These findings are partially consistent with previous findings that have demonstrated the association between aging and decreased trigeminal sensitivity [15]. On the other hand, the present data also support previous findings showing that older people tend to overrate their olfactory abilities [27].

For the psychophysical tests administered, the age‐based results were consistent with normative data on chemosensory function demonstrating decreases with age. Specifically, we found that being in the older age group was associated with significantly poorer performance on the three‐item odor identification test and significantly lower AmmoLa intensity ratings.

A major strength of the study was the diversity and number of participants (*N* = 1001) as the data were collected from visitors attending a science museum—and not a clinic. Hence, the data can be seen as a cross‐sectional study of the general population. Another strength of the study was the large number of quick subjective and psychophysical tests that were administered, providing a rich chemosensory profile for each participant. Related to our testing environment, the main limitation of the study was the inability to control the busy open public space in which the data were collected, and thus various ambient factors may have affected the participants' responses. However, the large number of participants tested helped to stabilize the response data, and all the measures were administered by the same researcher using a standardized protocol. Therefore, ambient factors are unlikely to have confounded our results. The use of self‐report measures was potentially another limitation of the current study; however, given the significant intercorrelations within the subjective and objective measures, convergent validity was established.

## 5 Conclusions

Our findings indicate that greater self‐reported tearing experienced when cutting onions is related to other self‐reported measures of chemosensory function, especially those related to trigeminal sensitivity. However, given the mixed findings with respect to age and the use of self‐report measures, the findings should be interpreted with caution. Moreover, the results must also be interpreted cautiously because any self‐report biases present in our data likely occurred across all the self‐report measures administered and their associated correlations. Though the relationship between onion‐induced tearing and trigeminal sensitivity is limited and is itself insufficient as a test of chemosensory function, it can provide valuable additional information in a clinical setting. Future research would benefit from conducting studies in controlled environments, where sensitivity to propanthialoxide and other compounds released from cutting onions is measured, along with self‐reported ratings and psychophysical tests of smell, taste, and trigeminal function.

## Funding

The authors have nothing to report.

## Conflicts of Interest

The authors declare no conflicts of interest.

## Data Availability

The data that support the findings of this study are available on request from the corresponding author. The data are not publicly available due to privacy or ethical restrictions.
